# Society Meetings

**Published:** 1911-09

**Authors:** 


### SOCIETY MEETINGS

THE American Association of Clinical Research will hold its third annual meeting on September 27th and 28th at Boston.

THE American Medical Editors' Association at the annual meeting in June elected the following officers for 1911-1912.

President, Surgeon-General Walter Wyman, Washington, D. C.; first vice-president, Thomas L. Stedman, M.D.; *Medical Record,* New York; second vice-president, Walter Lindley, M.D., *Southern California Practitioner,* Los Angeles; secretary and treasurer, Joseph MacDonald, Jr., M.D., *American Journal of Surgery,* New York.

Executive Committee: W. C. Abbott, M.D., Chicago, Ill., *American Journal of Clinical Medicine;* C. L. Stevens, M.D., Athens, Pa., *Pennsylvania State Medical Journal;* G. H. Kreidler, M.D., Cincinnati, Ohio, *Lancet and Clinic.*

The American Medical Association has appointed the following committee to draft a bill for presentation to the next congress, providing for the establishment of a national health deparment

Dr. Herman M. Biggs of New York city, Dr. W. A. Evans of Chicago, Dr. S. G. Dixon of Pennsylvania, Dr. J. N. Hutty of Indianapolis, Dr. J. N. McCormack of Kentucky, Dr. H. P. Wolcott of Cambridge, Mass., and Dr. W. C. Woodward of the District of Columbia. This committee will appoint a subcommittee to act in every state in the Union.

